# RNA‐dependent RNA polymerase 1 delays the accumulation of viroids in infected plants

**DOI:** 10.1111/mpp.13104

**Published:** 2021-07-23

**Authors:** Shuai Li, Zhixiang Zhang, Changyong Zhou, Shifang Li

**Affiliations:** ^1^ State Key Laboratory for Biology of Plant Diseases and Insect Pests Institute of Plant Protection Chinese Academy of Agricultural Sciences Beijing China; ^2^ Citrus Research Institute Chinese Academy of Agricultural Sciences/Southwest University Chongqing China; ^3^ Environment and Plant Protection Institute Chinese Academy of Tropical Agricultural Sciences Haikou China

**Keywords:** RDR1, resistance, RNA silencing, SA, salicylic acid, tobacco, tomato, viroid

## Abstract

RNA‐dependent RNA polymerase 1 (RDR1) is essential for plant antiviral defence, but its role in plant defence against viroid infection remains unknown. The present study aimed to identify the function and mechanism of RDR1 in plant resistance to viroid infection. Overexpression of *Nicotiana tabacum RDR1* (*NtRDR1*) delayed the accumulation of potato spindle tuber viroid (PSTVd) genomic RNA and PSTVd‐derived small RNA (sRNA) in *Nicotiana benthamiana* plants at the early invasion stage, but not in the late stage of infection. Conversely, virus‐induced gene silencing of tomato *RDR1* (*SlRDR1a*) increased the susceptibility to PSTVd infection (increased viroid accumulation). Salicylic acid (SA) pretreatment induced *SlRDR1a* expression and enhanced the defence against PSTVd infection in tomato plants. Our study demonstrated that RDR1 is involved in SA‐mediated defence and restricts the early systemic invasion by PSTVd in plants. The decreased PSTVd accumulation in *N*. *benthamiana* was not caused by efficient accumulation of PSTVd sRNAs. These results deepen our understanding of the mechanism of RDR1 in plant defence responses to viroid attack.

## Graphical abstract

Downregulation of RDR1a in tomato increased susceptibility to PSTVd infection; severe leaf curling with vein necrosis occurred in TRV:SlRDR1a tomato compared with TRV:00 tomatoes 28 days postinoculation (dpi) with PSTVd.

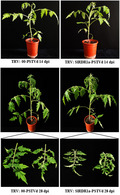

## 1 INTRODUCTION

RNA silencing is the main plant antiviral mechanism (Ding & Voinnet, 2007), in which RNA‐dependent RNA polymerase 1 (RDR1) is a key element in regulating plant viral resistance (Ahlquist, 2002; Qi et al., 2004; Willmann et al., 2011). *RDR1* was first cloned from tomato (*Solanum lycopersicon* 'Rutgers') infected by potato spindle tuber viroid (PSTVd) (Schiebel et al., 1998). *RDR1* transcription was induced in tomato (cv. Rentita) leaves systemically infected with PSTVd, and the RDR1 catalytic activity in leaves increased by about threefold compared with that in healthy leaf tissue (Schiebel et al., 1993). Virus infection also induces *RDR1* expression in other plants, such as *Arabidopsis thaliana*, tobacco, cucumber, maize, and pepper (Basu et al., 2018; He et al., 2010; Khan et al., 1986; Leibman et al., 2018; Qin et al., 2017; Xie et al., 2001; Yu et al., 2003). In addition, the expression of *RDR1* is induced by plant hormones such as salicylic acid (SA) (Xie et al., 2001; Yu et al., 2003) and can be modulated by plant microRNAs (Wang et al., 2016). Previous studies found that plants were more sensitive to virus infection when *RDR1* expression was repressed (Yu et al., 2003). In *Nicotiana tabacum NtRDR1* gene knockout mutants, inoculation with tobacco mosaic virus (TMV) resulted in higher accumulation of TMV genomic RNA and more severe symptoms compared with those in wildtype plants (Xie et al., 2001). Silencing of *NtRDR1* transcription using a double‐stranded, *NtRDR1‐*derived RNA hairpin resulted in increased accumulation of potato virus Y (PVY) RNA after infection (Rakhshandehroo et al., 2009).

Notably, the *Nicotiana benthamiana NbRDR1* gene has a 72 bp insertion that prematurely terminates the gene's translation. This disruption of translation implies a natural loss‐of function of the RDR1 in *N*. *benthamiana* that has been suggested to be the cause of the extreme susceptibility of *N*. *benthamiana* to a wide range of viruses, and makes these plants an excellent tool for plant molecular virologists (Bally et al., 2018; Yang et al., 2004). This phenomenon also verifies the antiviral function of RDR1 from another aspect. *N*. *benthamiana* plants transformed with *RDR1* genes from *Medicago truncatula* and pepper (*Capsicum* *annuum*) are more resistant to infection by TMV, turnip vein‐clearing virus, and sunn‐hemp mosaic virus (Qin et al., 2017; Yang et al., 2004). In addition, *RDR1* induction is involved in regulating symptom recovery after virus infection in tobacco (Basu et al., 2018). The antiviral properties of RDR1 have also been found in *A*. *thaliana* and cucumber. The *AtRDR1* knockout mutant accumulates higher and more persistent levels of viral RNAs in infected leaves than those in wildtype plants (Yu et al., 2003). Multiple *RDR1* genes are involved in virus resistance in cucumber and are regulated in a coordinated fashion with different expression profiles, for example constitutive expression of *RDR1* in a transgenic cucumber line (*Cucumis sativus*) causes broad resistance to potyviruses (Leibman et al., 2011). These studies confirmed that RDR1 plays an important role in plant antiviral defence. However, in some cases *RDR1* showed no significant correlation with plant antiviral defence. Silencing of the potato (*Solanum* *tuberosum*) *RDR1* gene (*StRDR1*) did not increase potato susceptibility when challenged with three viruses: PVY, potato virus X (PVX), and TMV (Hunter et al., 2016). Surprisingly, *N*. *benthamiana* transformed with *RDR1* from *N*. *tabacum* (*NtRDR1*) exhibit hypersusceptibility to plum pox virus and other viruses (Ying et al., 2010). This highlights the complexity of plant antiviral defence mechanisms. Plants must have different antiviral regulation genes and pathways that might interact and cooperate with each other. For example, reduced accumulation of NtRDR1 in *N*. *tabacum* results in lower expression of other antiviral defence‐related genes after PVY infection, such as RNA‐dependent RNA polymerase 6 (RDR6), which is involved in RNA silencing (Rakhshandehroo et al., 2009). Consequently, to fully determine the antiviral function of RDR1, we need to explore its molecular mechanism. *Arabidopsis* mutants lacking *RDR1* produce lower levels of virus‐derived small interfering RNAs (siRNAs), making them more susceptible to turnip mosaic virus (TuMV) and CMV infection. This suggests that RDR1 enhances plant viral resistance by amplifying virus‐derived siRNAs, thus enhancing plant RNA silencing (Garcia‐Ruiz et al., 2010; Wang et al., 2010). Virus infection triggers widespread silencing of *Arabidopsis* genes by producing abundant endogenous siRNAs in plants, and this broad‐spectrum host antiviral activity depends on *RDR1* expression (Cao et al., 2014). RDR1 also regulates plant genes involved in hormone synthesis and DNA methylation, suggesting that RDR1 may regulate plant antiviral defences in multiple ways (Lam et al., 2012; Stroud et al., 2013; Wang et al., 2014). Thus, RDR1 not only regulates plant viral resistance, but also acts as an intersecting node of plant gene expression regulatory pathways, such as RNA silencing and signal transduction.

In comparison with plant viruses, we know little about *RDR1’*s involvement in the interaction between viroids and their hosts. Only a few viroids are known to induce *RDR1* expression in plants (Campos et al., 2014; Schiebel et al., 1998; Xia et al., 2017). Therefore, it is necessary to study the role and mechanism of RDR1 in plant antiviroid defence. Our previous study showed that infection with hop stunt viroid (HSVd) induces *RDR1* expression in cucumber (*Cucumis sativus*) (Xia et al., 2017); however, whether *RDR1* induction is a common phenomenon in plants responding to viroid infection is unclear. Consequently, we aimed to use combinations of PSTVd and tomato/tobacco to identify the function and mechanism of RDR1 in plant resistance to viroid infection. This study will improve our understanding of viroid–host interactions and provide new ideas and methods to prevent and control viroid diseases.

## 2 RESULTS

### 2.1 *NtRDR1* overexpression delayed PSTVd accumulation in *N*. *benthamiana*

**FIGURE 1:**
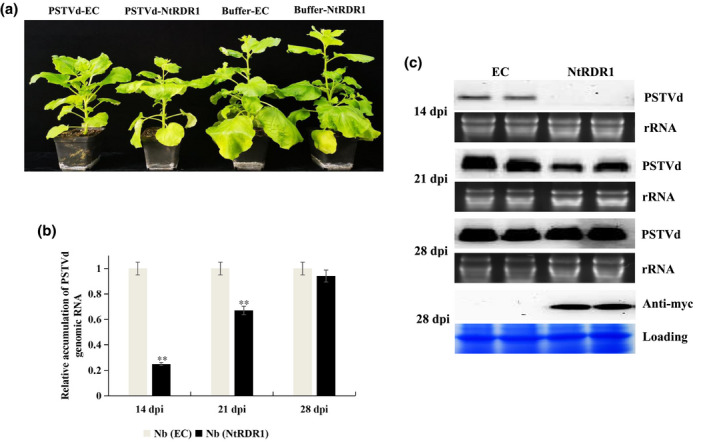
Time‐course analysis of accumulation levels of potato spindle tuber viroid (PSTVd) genomic RNA in transgenic *Nicotiana benthamiana* plants. (a) PSTVd or phosphate buffer (mock) was inoculated to the lower leaves of empty vector control (EC) and *N. tabacum* RNA‐dependent RNA polymerase 1 (*NtRDR1*) transformed plants. Pictures were taken at 28 days postinoculation (dpi). (b) Accumulation of PSTVd genomic RNA was analysed using quantitative reverse transcription PCR (RT‐qPCR). Experiments were performed in triplicate and the data are expressed as the mean ± *SD*. Error bars indicate the *SD* of three biological replicates of RT‐qPCR analysis. **p < 0.01 using Student's *t* test. (c) Time‐course accumulation of PSTVd genomic RNA. Northern blot detection with digoxigenin (DIG)‐labelled PSTVd complementary RNA (cRNA) probe in NtRDR1 and EC transgenic lines at 14, 21, and 28 dpi. As a loading control, ribosomal RNAs (rRNAs) were stained with ethidium bromide. Western blotting detection of myc‐NtRDR1 with anti‐myc antibodies in NtRDR1 and EC transgenic lines. Coomassie blue‐stained total proteins are shown as loading controls

To confirm that the *NbRDR1m* gene expressed a transcript containing the 72‐nucleotide (nt) insert compared with *NtRDR1*, reverse transcription PCR (RT‐PCR) was performed on RNA isolated from transgenic *N*. *benthamiana* NtRDR1 line (which expressed NtRDR1‐myc fusion protein) and control *N*. *benthamiana* line (empty vector transgenic, EC) using NbRDR1/NtRDR1 primers (Table S1) (Ying et al., 2010). The results showed that the EC samples contained a single amplicon, whereas the NtRDR1 samples contained two amplicons that differed by approximately 72 nt (Figure S1). Western blotting of myc‐NtRDR1 in transgenic *N*. *benthamiana* showed high amounts of myc‐NtRDR1 in the total protein extracted from NtRDR1 leaf tissue, but none in the EC tissues (Figure 1c, bottom panel). The results indicated that *NtRDR1* could be transcribed and translated in transgenic *N*. *benthamiana*, and the NtRDR1 plants did not show a significant difference in growth or phenotype compared with EC plants (Figure 1a). The confirmed NtRDR1 transgenic seedlings were used in subsequent experiments.

To investigate the potential role of RDR1 in defence against PSTVd infection, we first determined the relative accumulation of PSTVd genomic RNAs in *N*. *benthamiana* EC and NtRDR1 transgenic plants. Lower leaves of EC and NtRDR1 transgenic plants were inoculated mechanically with in vitro transcribed PSTVd RNA. The vegetative phenotypes were photographed at 14, 21, and 28 days postinoculation (dpi). PSTVd induced dwarfing symptoms in *N*. *benthamiana* (EC) compared with buffer‐treated EC plants; however, there was no significant phenotypic difference between PSTVd‐infected EC and NtRDR1 plants at 28 dpi (Figure 1a). Quantitative real‐time RT‐PCR (RT‐qPCR) assays revealed lower accumulation of PSTVd RNAs in NtRDR1 transgenic plants at 14 dpi (approximately 22% of that in EC plants); however, the difference narrowed at 21 dpi (about 65%) and no significant difference in PSTVd accumulation was observed between EC and NtRDR1 plants at 28 dpi (Figure 1b). Moreover, the results of northern blotting were similar to those of the RT‐qPCR assays. The intensity of PSTVd genomic RNA signals was lower in NtRDR1 transgenic plants than in EC plants at 14 dpi, but this intensity difference was almost indistinguishable at 28 dpi (late infection stage) (Figure 1c).

To gain insights into the function of other RDR1s in PSTVd infection, transgenic *N*. *benthamiana* plants expressing the homologous gene of *RDR1* from cucumber fused with the green fluorescent protein gene *GFP* were generated. Western blot hybridization confirmed the expression of CsRDR1c1‐GFP fusion protein in CsRDR1c1 transgenic lines (Figure S2, bottom panel). Subsequently, we determined the accumulation of PSTVd in infected EC and CsRDR1c1 transgenic plants. Northern blotting revealed that PSTVd RNA accumulation was significantly lower in CsRDR1c1 transgenic plants than in EC plants at 14 dpi, but this difference was almost indistinguishable at 28 dpi. Western blotting confirmed the expression of CsRDR1c1‐GFP in CsRDR1c1 transgenic plants but not in EC plants (Figure S2). These results demonstrated that NtRDR1 and CsRDR1c1 are involved in a defence pathway that restricts the early systemic invasion of PSTVd in *N*. *benthamiana*.

### 2.2 *NtRDR1* overexpression decreased the accumulation of PSTVd‐derived small RNA (sRNA) in *N. benthamiana*

**FIGURE 2:**
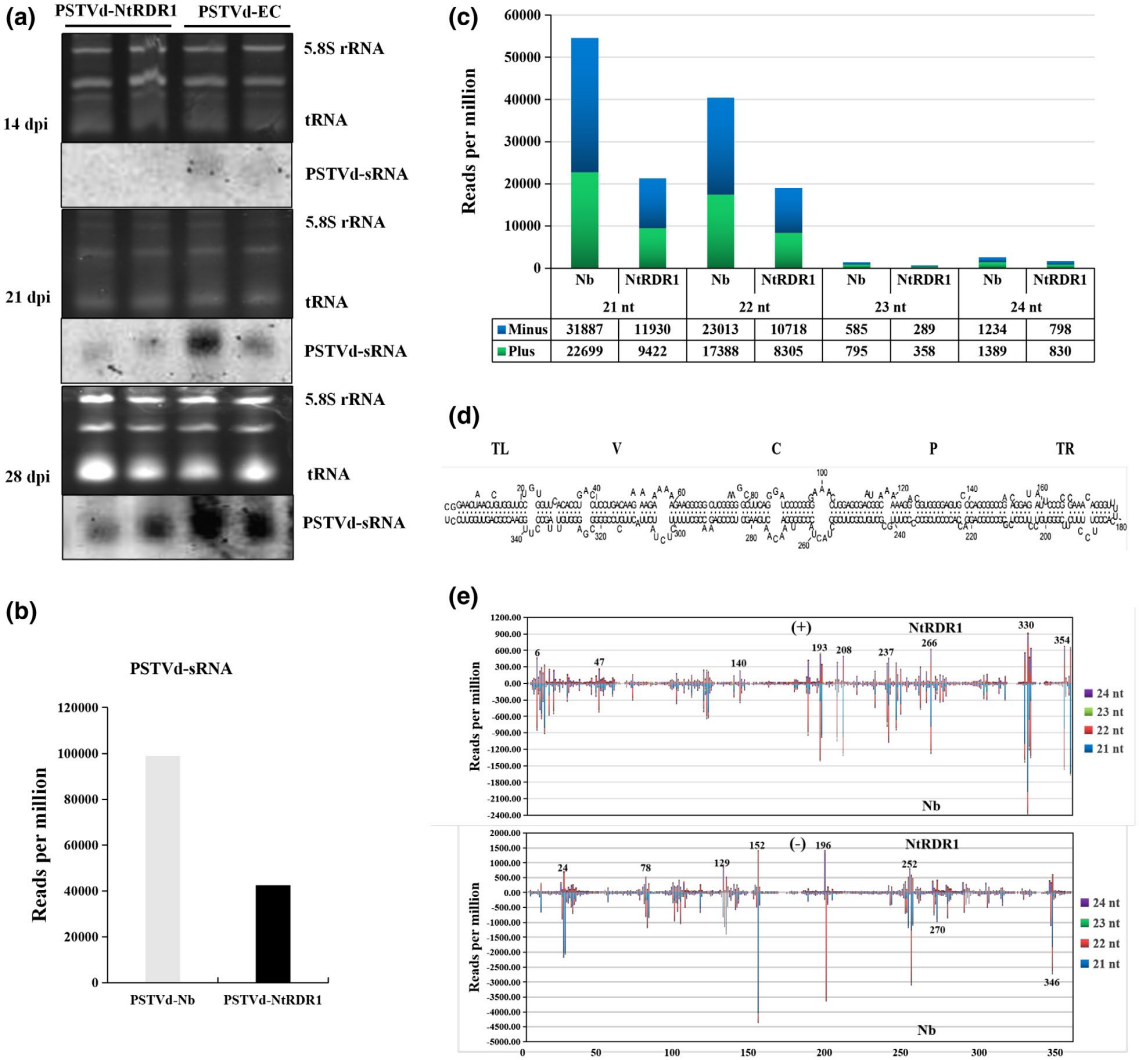
Accumulation of potato spindle tuber viroid (PSTVd) small RNAs (sRNAs) and the characterization of PSTVd sRNA populations in transgenic *Nicotiana benthamiana* plants. (a) Accumulation of PSTVd sRNAs was analysed by northern blotting with a digoxigenin (DIG)‐labelled PSTVd complementary RNA (cRNA) probe in *N. tabacum* RNA‐dependent RNA polymerase 1 (NtRDR1) and empty control (EC) transgenic lines at 14, 21, and 28 days postinoculation (dpi). Loadings were equalized by the signal intensity of 5.8S ribosomal RNA (rRNA) and transfer RNA (tRNA) stained with ethidium bromide. More PSTVd sRNAs derived from PSTVd genomic RNA accumulated in EC plants than in NtRDR1 plants. (b) The total number of PSTVd sRNAs reads derived from PSTVd‐EC lines (98,990 reads per million [RPM]) was about two‐fold higher than that in the PSTVd‐NtRDR1 lines (42,650 RPM). (c) Multiple PSTVd sRNAs in EC and *NtRDR1* plants. Histograms compare the size distribution of 21‐ to 24‐nt PSTVd sRNAs counts and the total counts of plus‐ and minus‐strand PSTVd sRNAs obtained by deep sequencing from PSTVd‐infected EC and NtRDR1 samples at 21 dpi. (d) Primary and rod‐like secondary structure of minimal free energy predicted for the plus‐strand of PSTVd. The relative positions of the terminal left (TL), pathogenic (P), central (C), variable (V), and terminal right (TR) domains are indicated. (e) Sequence profiles of PSTVd sRNAs derived from the genomic (+, upper panel) and anti‐genomic (−, lower panel) strands in EC and NtRDR1 plants. sRNA distribution profiles represent the sum of 21–24 nt PSTVd sRNA populations originating from EC and NtRDR1 plants infected with PSTVd mapped along the linearized PSTVd genome (nucleotides 1–357 from left to right)

In plants, RDR‐mediated production of siRNAs is important to enhance the effect of RNA silencing defence against plant viruses (Wassenegger & Krczal, 2006). In our study, PSTVd genomic RNA accumulation was suppressed in transgenic NtRDR1 *N*. *benthamiana* plants. Whether the suppressed accumulation of PSTVd genomic RNA correlates with the production of PSTVd sRNA is unknown; therefore we analysed the accumulation of PSTVd sRNA in PSTVd‐infected EC and NtRDR1 transgenic lines at 14, 21, and 28 dpi. At 14 dpi, a marked intensity of the PSTVd sRNA signals was observed in the EC lines but not in the NtRDR1 lines. Increasing accumulation of PSTVd sRNA was observed in the NtRDR1 lines at 21 and 28 dpi, which was always lower than in the EC lines (Figure 2a). Thus, the accumulation of PSTVd sRNA correlated positively with that of PSTVd genomic RNA (Figure 2a) and suggested that the decreased PSTVd accumulation in the *N*. *benthamiana* NtRDR1 transgenic line was not caused by efficient accumulation of PSTVd sRNA.

To further characterize the PSTVd sRNA populations, high‐throughput sequencing of sRNAs was performed for EC and NtRDR1 lines infected with PSTVd at 21 dpi, when RNA gel blot hybridization revealed conspicuous differences in the PSTVd genomic and viroid‐derived sRNA between EC and NtRDR1 plants. The obtained sRNA sequences were mapped to PSTVd genome RNA and most of the PSTVd sRNAs were 21–24 nucleotides (nt) in length. The mapped sequences between 21 and 24 nt were pooled, and each set of sequences was BLAST searched against the nucleotide sequences of PSTVd strains, with no mismatches allowed. The total number of reads derived from PSTVd‐EC lines (98,990 reads per million [RPM]) was about two‐fold higher that in the PSTVd‐NtRDR1 lines (42,650 RPM) (Figure 2b), which presumably reflected the initial PSTVd sRNA concentration, being lower in the NtRDR1 lines than in the EC lines, and was consistent with PSTVd sRNA blotting results (Figure 2a). In all samples, the number of reads derived from the genomic (+) strand of PSTVd was lower than that from anti‐genomic (−) strand (Figure 2c). In addition, rearranging our data according to read length revealed that the 21 nt RNAs were the largest class in the EC and NtRDR1 lines, followed by 22 and 24 nt RNAs, indicating the involvement of multiple Dicer‐like (DCL) endonucleases in PSTVd sRNA biogenesis in *N*. *benthamiana* (Figure 2c). To compare the specific hot‐spot profiles of the PSTVd sRNA, the 5′‐termini of the plus strand and the 3ʹ‐termini of the minus strand generated PSTVd sRNA reads, both containing 21 to 24 nt, were mapped to the corresponding positions on the PSTVd genomic and anti‐genomic RNA, and the frequency (RPM) was determined (Table S2). The sRNA distributions for PSTVd from each genome position in the genomic and anti‐genomic strands was biased toward several positions (hot spots), such as 193–266 in the genomic strand and positions 152, 196, 252, and 346 in the anti‐genomic strand. Comparison of NtRDR1 and lower EC data revealed that the overall sRNA distribution profiles in the genomic strand were almost identical. Although the hot‐spot patterns formed by the anti‐genomic PSTVd sRNAs were mostly similar in the EC and NtRDR1 samples, on careful comparison there were some differences at some positions. Compared with NtRDR1, EC produced more sRNAs at positions 23, 25, 79, 130, and 197, especially the 21‐nt and 22‐nt sRNAs. In addition, positions 196 and 252 were enriched in sRNAs in the NtRDR1 population with respect to EC (Figure 2d,e, Table S2). However, the differences in the sRNA hot‐spot patterns on both PSTVd strands indicated the existence of some unknown factors determining these differential profiles.

### 2.3 Downregulation of *RDR1a* in tomato increased susceptibility to PSTVd infection

**FIGURE 3:**
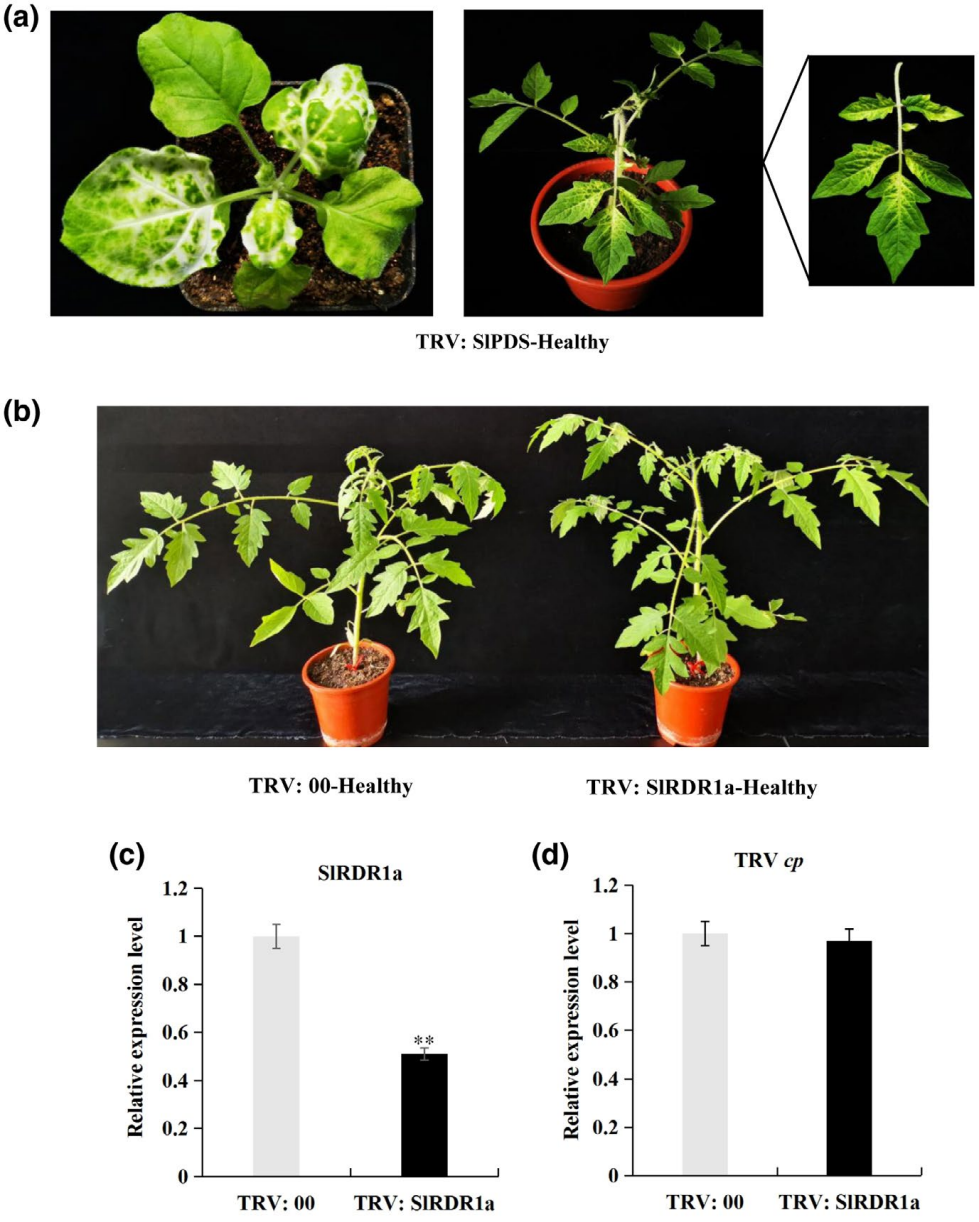
Silencing of the tomato RNA‐dependent RNA polymerase 1 gene (*SlRDR1a*) in cv. Rutgers tomato lines exhibited no clear difference of phenotypes compared with control plants. Tobacco rattle virus (TRV):00 (negative control), plants agroinfiltrated with pTRV2‐EV (empty vector) along with pTRV1; pTRV:SlPDS (tomato phytoene desaturase [*Sl* *PDS*] gene), plants agroinfiltrated with pTRV2:SlPDS along with pTRV1; pTRV:SlRDR1a, plants agroinfiltrated with pTRV2‐SlRDR1a along with pTRV1. Each experiment was performed at least three times with true biological replicates. (a) pTRV: SlPDS was used as a positive control for the virus‐induced gene silencing (VIGS) experiment because knockdown of *Sl* *PDS* results in bleached leaves. The system leaves of *Nicotiana benthamiana* and tomato plants exhibited a bleached phenotype at 14 days postinoculation (dpi). (b) At 28 dpi, the plants exhibited no clear phenotypic difference between *SlRDR1a*‐silenced plants and negative control plants. (c) Quantitative reverse transcription PCR (RT‐qPCR) of total RNA extracted from systemic leaf samples from the agroinfiltrated plants to evaluate the downregulation of *Sl* *RDR1a* in tomato plants. **p < 0.01 by Student's *t* test. (d) The expression levels of TRV capsid protein coding gene (*cp*) in SlRDR1a and TRV:00 tomato plants, as measured using RT‐qPCR

Tomato can be infected with PSTVd, and infected tomatoes exhibit severe leaf curling with vein necrosis and plant dwarfing, seriously threatening tomato fruit production (Diermann et al., 2010). Among the identified RDRs in *S*. *lycopersicon*, SlRDR1a shared the highest amino acid sequence (86%) identity with NtRDR1 (Liao et al., 2014). Despite this high sequence homology between SlRDR1a and NtRDR1, little is known about the roles of SlRDR1a in the tomato response to PSTVd infection (Figure S3). Therefore, we explored the function of SlRDR1a in tomato. First, tobacco rattle virus (TRV)‐induced gene silencing was used to suppress *SlRDR1a* expression in tomato. pTRV1, along with pTRV2:SlRDR1a, was transformed into *Agrobacterium* *tumefaciens*, which was used to agroinfiltrate tomato seedlings. pTRV2:SlPDS (to silence the phytoene desaturase [*Sl* *PDS*] gene) served as a positive control for a successful viral infection and agroinfiltration of empty pTRV2 vector (pTRV2: EV) served as a negative control. At approximately 14 dpi, the pTRV2‐SlPDS tomatoes and *N*. *benthamiana* exhibited bleached leaves (Figure 3a). Phenotypically, the tomatoes agroinfiltrated with pTRV2‐SlRDR1a did not exhibit obvious abnormalities compared with the pTRV2‐EV‐inoculated tomatoes (Figure 3b). Total RNA extracted from systemic leaf samples from the agroinfiltrated plants at 14 dpi was subjected to RT‐­qPCR, which showed that the expression level of *SlRDR1a* in the silenced plants was 50% of that in the pTRV2‐EV‐inoculated tomatoes (Figure 3c). Semiquantitative RT‐PCR was also performed to confirm *SlRDR1a* silencing in tomatoes (Figure S4). The TRV accumulation levels were similar between the *SlRDR1a*‐silenced and control tomatoes (Figure 3d). The data showed that silencing *SlRDR1a* did not cause any morphological changes to the tomato plants.

**FIGURE 4:**
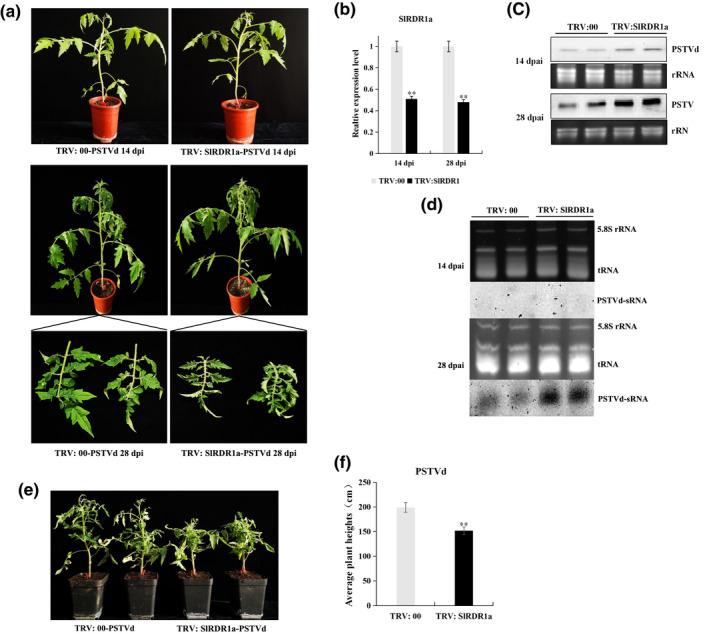
Silencing of the tomato RNA‐dependent RNA polymerase 1 gene (*SlRDR1a*) impaired the tolerance of cv. Rutgers tomato to potato spindle tuber viroid (PSTVd) infection. Phenotypes of tobacco rattle virus (TRV):00 and TRV:SlRDR1a Rutgers tomato plants. (a) Tomato plants were agroinfiltrated with pTRV2‐EV and pTRV2‐SlRDR1a in the lower leaves. Fourteen days later, pCAM2300‐PSTVd was inoculated into TRV:00 and TRV:SlRDR1a plants. Pictures were taken at 14 (upper) and 28 (lower) days postinoculation (dpi). (b) The expression levels of *SlRDR1a* mRNA in TRV:00 and TRV:SlRDR1a plants were analysed using quantitative reverse transcription PCR (RT‐qPCR) at 14 and 28 dpi. **p < 0.01 by Student's *t* test. (c) Suppression of *SlRDR1a* favours the accumulation of PSTVd in Rutgers tomato. Samples were collected from the upper uninoculated leaves of tomato plants at 14 and 28 dpi with PSTVd in the lower leaves. The accumulation levels of PSTVd genomic RNA at 14 and 28 dpi were analysed using northern blotting. Ribosomal RNAs (rRNAs) were stained with ethidium bromide as a loading control. The results are based on three biological replicates collected from six individual plants. (d) Accumulation of PSTVd sRNA was analysed using northern blotting at 14 and 28 dpi. Loadings were equalized by the signal intensity of 5.8S rRNA and tRNA stained with ethidium bromide. (e) PSTVd‐induced phenotypes in TRV:00 and TRV:SlRDR1a plants. At 50 dpi, *SlRDR1a* knockdown plants (right) infected with PSTVd showed severe stunting and curled leaves with respect to PSTVd‐inoculated controls plants (left). The average heights of PSTVd‐infected TRV:00 and TRV:SlRDR1a plants were measured at 50 dpi (f). The results are expressed as the mean ± *SD*, *n* = 6, **p < 0.01 by Student's *t* test

Tomato plants were agroinfiltrated with TRV:00, TRV:SlRDR1a, or TRV:SlPDS in the lower leaves. At approximately 14 dpi, when the TRV:SlPDS agroinfiltrated tomatoes started to show bleaching, the lower leaves of TRV:SlRDR1a or TRV:00 plants were agroinfiltrated with pCAM2300‐PSTVd. The vegetative phenotypes were photographed at 14 and 28 dpi after PSTVd infection. At 14 dpi, PSTVd‐infected TRV:00 tomato plants were asymptomatic, but the systemic leaves of the *SlRDR1a* knockdown plants (TRV:SlRDR1a) developed moderate leaf curling. Subsequently, both TRV:00 and TRV:SlRDR1a plants developed a leaf curling phenotype in systemic leaves, while severe leaf curling with vein necrosis occurred in TRV:SlRDR1a plants compared with TRV:00 tomatoes at 28 dpi (Figure 4a). To confirm the suppression of *SlRDR1a* expression and evaluate the effect of SlRDR1a on PSTVd accumulation in tomatoes, leaf samples were collected from the upper uninoculated leaves at 14 and 28 dpi, and RNA was extracted. RT‐qPCR confirmed the suppression of *SlRDR1a* in the TRV:SlRDR1a‐PSTVd plants (Figure 4b). Northern blotting using digoxygenin (DIG)‐labelled PSTVd cRNA probes showed higher accumulated levels of PSTVd genomic RNA in the *SlRDR1a* knockdown plants than in the control plants at 14 and 28 dpi (Figure 4c). PSTVd sRNA accumulation was also analysed using northern blotting in TRV:00 and TRV:SlRDR1a plants at 14 and 28 dpi. At 14 dpi, intense signals of PSTVd‐sRNA were observed in both lines. At 28 dpi, the TRV:SlRDR1a plants showed higher levels of PSTVd‐sRNA compared with that in TRV:00 plants (Figure 4d). Thus, the accumulation of PSTVd sRNAs mirrored that of PSTVd genomic RNA accumulation in tomatoes. Importantly, in the late stage of PSTVd infection (50 dpi), PSTVd‐inoculated TRV:SlRDR1a tomatoes showed severe stunting and curled leaves compared with those of PSTVd‐inoculated TRV:00 plants (Figure 4e). The average height of the *SlRDR1a* knockdown plants was significantly lower than that of the TRV:00 plants (Figure 4f). Altogether, the results showed a clear correlation between the *SlRDR1a* mRNA abundance and the severity of the PSTVd phenotype in tomatoes lines. *SlRDR1a* mRNA suppression increased tomato susceptibility to PSTVd infection, resulting in increased viroid accumulation.

### 2.4 RDR1 is involved in SA‐mediated defence against PSTVd infection

**FIGURE 5:**
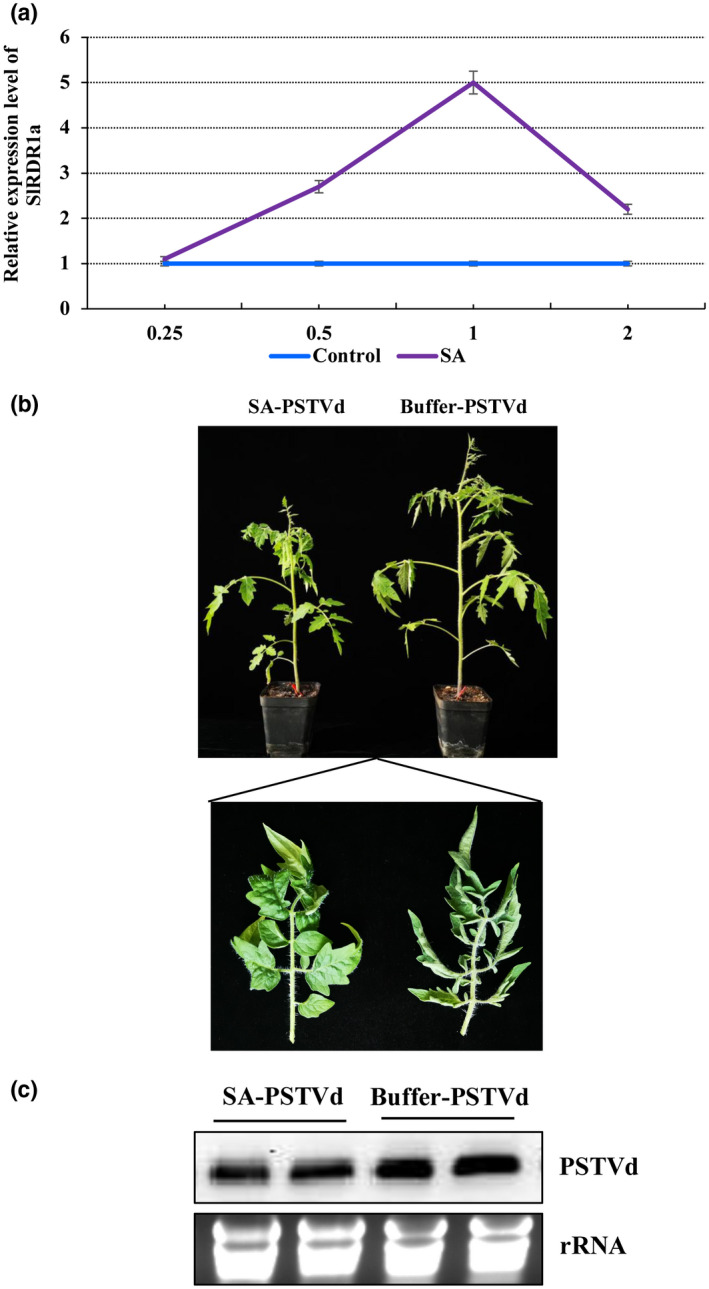
Exogenous application of salicylic acid (SA) can improve the resistance of tomato to potato spindle tuber viroid (PSTVd) infection. (a) Time course of the changes in the expression of tomato RNA‐dependent RNA polymerase 1 (*SlRDR1a*) transcripts afer SA treatment of tomato plants. Samples were collected from the upper leaves of tomato plants after SA or buffer treatment at 0.25, 0.5, 1, and 2 days, respectively. The results are presented as the mean ± *SD*, *n* = 6. (b) SA can compromise the symptoms of PSTVd in tomato. Phenotypes of PSTVd infection after SA or buffer pretreatment of tomato plants. Tomato leaves were sprayed with 2 mM SA or buffer 24 hr before PSTVd inoculation. Pictures were taken at 28 days postinoculation (dpi). The results are expressed as the mean ± *SD*, *n* = 6. (c) SA can delay the accumulation of PSTVd in tomato. Samples were collected from the upper uninoculated leaves of tomato plants and at 28 dpi. Ribosomal RNAs (rRNAs) were stained with ethidium bromide as loading controls. These results are based on three biological replicates collected from six individual plants

The signalling molecule salicylic acid (SA) plays important roles in both compatible and incompatible plant interactions with pathogens, including plant viruses (Dempsey et al., 1999). SA plays an important role in plant antiviral defence and RDR1 is involved in the SA‐induced defence response to virus infection (Liao et al., 2014; Yang et al., 2004). A previous study showed that citrus exocortis viroid (CEVd) infection in tomato induces the expression of *ToRDR1* and also other RNA silencing‐related genes such as *ToDCL1*, *ToDCL2*, *ToDCL4*, and *ToRDR2*, with the exception of *ToRDR6*. Also in that study, SA treatments induced the expression of RNA silencing‐related genes such as *ToDCL1*, *ToDCL2*, *ToRDR1*, and *ToRDR2* in tomato, resulting in resistance to tomato mosaic virus (ToMV) in tomato (Campos et al., 2014). Therefore, we further explored the SlRDR1a induction and SA signal transduction relationship in tomatoes during PSTVd infection. Tomato leaves were sprayed with 2 mM SA or buffer (control) on both the adaxial and abaxial surfaces. Time course changes in *SlRDR1a* transcription were measured using RT‐qPCR (Figure 5a). *SlRDR1a* expression began to increase at 6 hr postinoculation (hpi), and reached a maximum induction of 5.1‐fold at 24 hpi compared with control plants; however, at 24 hpi, the expression level decreased rapidly. To determine the relationship between SA and the defence response against PSTVd infection in tomato plants, the foliage was sprayed until runoff with 2 mM SA or buffer daily for 3 days, and the lower two fully developed leaves were used for agroinfiltration with pCAM2300‐PSTVd.

**FIGURE 6:**
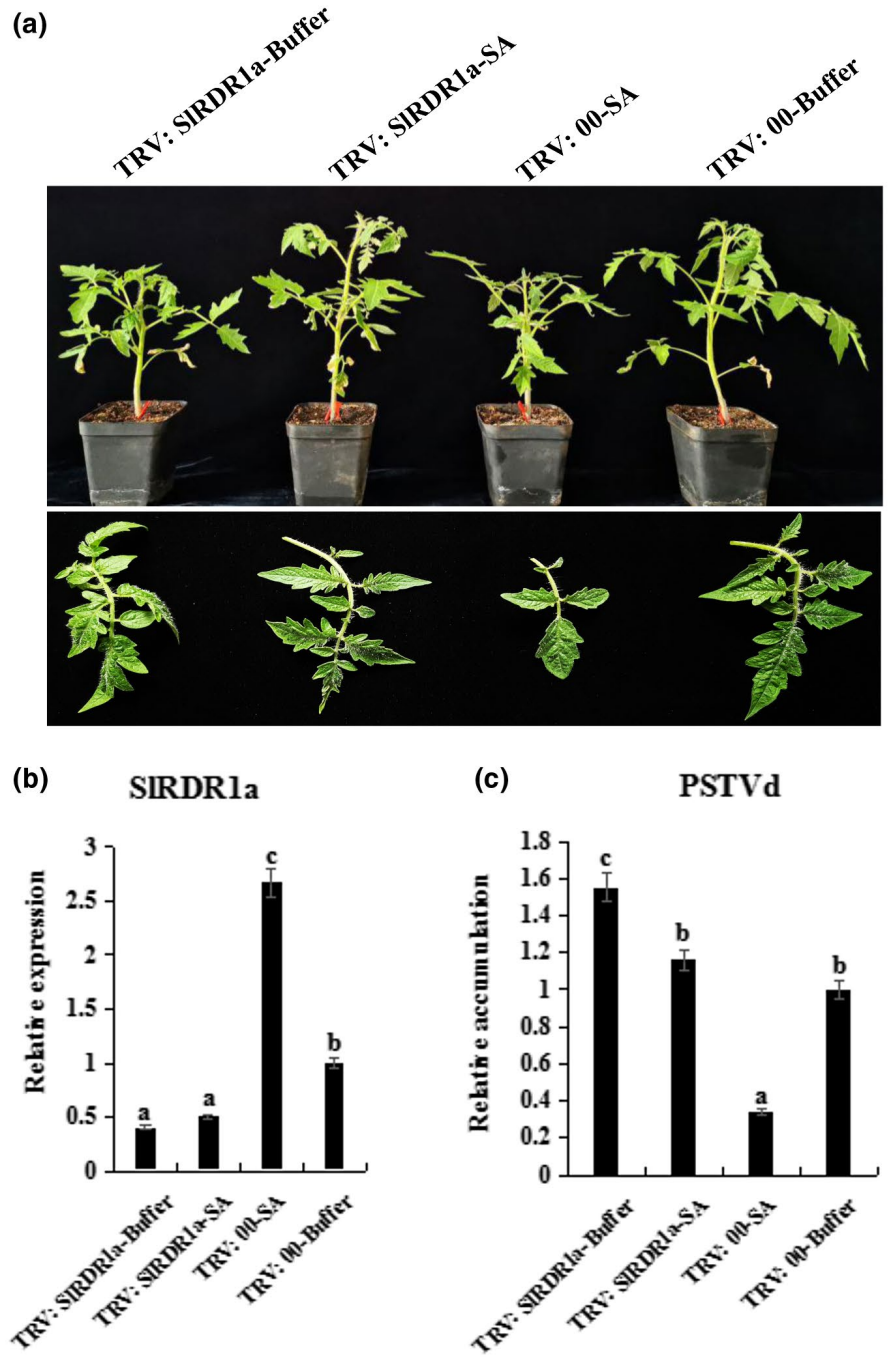
The resistance of RNA‐dependent RNA polymerase 1 (*SlRDR1a*)‐silenced tomato to potato spindle tuber viroid (PSTVd) can be partially restored by exogenous application of salicylic acid (SA). (a) Effects of PSTVd infection and SA application in *SlRDR1a*‐silenced tomato plants. Pictures were taken at 21 days postinoculation (dpi) with PSTVd. Tobacco rattle virus (TRV):00 and TRV:SlRDR1a tomato leaves (14 days later) were sprayed with 2 mM SA or buffer 24 hr before PSTVd inoculation. (b, c) Total RNA extracted from uninoculated leaves of tomato plants was subjected to quantitative reverse transcription PCR (RT‐qPCR) to evaluate the relative expression of *SlRDR1a* mRNA at 4 dpi and the relative accumulation of PSTVd genomic RNA at 21 dpi

At 28 dpi, PSTVd caused more severe leaf curling of the systemic leaves in the no‐treated controls than in the SA‐pretreated plants (Figure 5b). The newly developed systemic leaves were collected simultaneously for northern blotting experiments, which showed lower accumulation of PSTVd genomic RNA in the SA‐PSTVd plants than in the controls at 28 dpi (Figure 5c). These results suggest that SA induction of *SlRDR1a* in Rutgers tomato enhanced resistance to PSTVd infection. We then investigated the effects of SA pretreatment on PSTVd infection in *SlRDR1a*‐silenced tomato plants. TRV:00 and TRV:SlRDR1a tomato leaves (at 14 dpi) were pretreated with 2 mM SA or buffer. After 24 hr, the lower leaves were agroinfiltrated with PSTVd. The vegetative phenotypes were photographed and measured at 21 dpi. Simultaneously, total RNA was extracted from noninoculated upper leaves of tomato plants and subjected RT‐qPCR assay to evaluate the relative expression of *SlRDR1a* (4 dpi) and the relative accumulation of PSTVd genomic RNA (21 dpi) in tomato plants inoculated with PSTVd. The leaves failed to display any readily observable phenotype in TRV:00‐SA tomato plants. In contrast, TRV:SlRDR1a‐Buffer plants showed substantially increased PSTVd susceptibility and visible damage, with curling and crinkling of leaves. TRV:SlRDR1a‐SA plants showed significantly decreased PSTVd susceptibility, with a phenotype similar to that of the TRV:00‐Buffer plants (Figure 6a). Compared with that in TRV:00‐Buffer control plants, *SlRDR1a* transcription increased significantly in PSTVd‐inoculated TRV:00‐SA plants at 4 dpi. However, the *SlRDR1a*‐silenced plants exhibited significantly lower levels of *SlRDR1a* transcription under both SA and buffer pretreatment (Figure 6b). Moreover, PSTVd genomic RNA levels in noninoculated upper leaves at 21 dpi correlated with the observed symptoms (Figure 6c). Compared with TRV:00‐Buffer plants challenged with PSTVd, TRV:SlRDR1a‐Buffer plants had a 55% increase in PSTVd RNA, SA‐pretreated TRV:00 plants showed a 70% decreases in PSTVd RNAs, and TRV:SlRDR1a‐buffer plants showed no significant difference in PSTVd accumulation.

Thus, SA pretreatment could not increase *SlRDR1a* transcription in *SlRDR1a*‐silenced plants, whereas exogenous SA could partially restore PSTVd resistance of SlRDR1a‐silenced tomato plants. Our results provide evidence for crosstalk between the SlRDR1a and the SA pathway in response to PSTVd infection.

## 3 DISCUSSION

Viroids, the smallest infectious agents endowed with autonomous replication, are single‐stranded circular RNAs (about 250–400 nt) without protein‐coding ability. Despite their simplicity, they infect and cause diseases in economically relevant herbaceous and woody plants (Flores et al., 2015, 2017).

To defend against viroid pathogens, plants have developed various strategies. DCL, Argonaute, and RDR proteins are key factors in plant defence against viroid infection. Previous reports suggested that members of *Pospiviroidae* and *Avsunviroidae* can be processed by DCL proteins in plants (Papaefthimiou et al., 2001). The synergism of *DCL2* and *DCL3* in *N*. *benthamiana* strongly suppressed PSTVd accumulation (Katsarou et al., 2016). In addition, suppression of *RDR6* favoured the accumulation of PSTVd in *N*. *benthamiana* and allowed PSTVd invasion of the stem cells of the shoot apical meristem (Adkar‐Purushothama & Perreault, 2019; Di Serio et al., 2010). Thus, the role and mechanism of these proteins in plant defence against viroid infection is pivotal in viroid research.

Among the eukaryotic RNA‐dependent RNA polymerase family, RDR1 is necessary for plant antiviral defence through RNA silencing, in which it is believed to amplify dsRNA templates (Cao et al., 2014). In our study, PSTVd infection induced the conspicuous expression of *SlRDR1a* in tomato plants (Figure S5); however, the role of RDR1 in the plant defence response to viroid infection remains poorly known. We used combinations of viroids with tomato or tobacco plants to identify RDR1’s function and mechanisms in plant viroid resistance. First, we determined the effect of varying *RDR1* expression on PSTVd infection. *N*. *tabacum NtRDR1* and *C. sativus* *CsRDR1c1* were overexpressed in *N*. *benthamiana*, followed by viroid inoculation and the detection of viroid and viroid‐derived sRNAs. The results show that NtRDR1 and CsRDR1c1 are involved in a defensive pathway that restricts the early systemic invasion of PSTVd in *N*. *benthamiana*; however, RDR1‐mediated resistance was not significant in the late infection stage. Second, the tomato RDR1 gene, *SlRDR1a*, was downregulated using virus‐induced gene silencing (VIGS). The *SlRDR1a*‐silenced tomatoes did not exhibit any morphological changes compared with wildtype plants (Figure 4); however, the *SlRDR1a*‐silenced plants were more susceptible to PSTVd infection, in which there was an inverse relationship between PSTVd accumulation and *SlRDR1a* transcript levels. Northern blotting experiments revealed a strong correlation between PSTVd RNA accumulation and the severity of PSTVd symptoms (Figure 5).

Previous research reported that the accumulation of PSTVd was suppressed in transgenic *SlRDR6* knockdown tomatoes, and the accumulation of PSTVd‐derived sRNAs in tomato plants correlated positively with that of PSTVd genomic RNA (Naoi et al., 2020). In our study, sRNA sequencing was used to confirm the accumulation of PSTVd sRNAs and identified specific RDR1‐amplified sRNAs in PSTVd‐infected EC and NtRDR1 plants. PSTVd sRNA accumulation correlated positively with PSTVd genomic RNA accumulation in *N*. *benthamiana* and tomato plants, suggesting that the decreased PSTVd accumulation in *N*. *benthamiana* was not caused by accumulation of PSTVd sRNAs (Figure 2). Thus, RDR1 does not regulate plant antiviroid defence by amplifying PSTVd dsRNA templates, but we cannot exclude the possibility that RDR1 could involved be in RNA silencing mechanisms regulating host RNAs. Therefore, further research is needed in subsequent work.

RDR1 regulates plant genes involved in hormone synthesis. Signal transduction via plant hormones is an important pathway to regulate plant growth, development, and metabolism, suggesting that RDR1 might also regulate antiviral defence via hormones (Lam et al., 2012). SA plays a critical role in plant defence against pathogen attack, mainly via two pathways: RDR1‐mediated RNA silencing and the alternative oxidase (AOX)‐associated defence pathway. The application of an exogenous AOX activator on tomato plants markedly induces the accumulation of *SlRDR1* and *SlAOX1a* transcripts and reduces TMV RNA accumulation, indicating that RDR1 is involved in the AOX‐mediated defence pathway against TMV infection (Liao et al., 2014). Furthermore, exogenous SA application on *N*. *tabacum* induces rapid nitric oxide (NO) accumulation, which functions upstream of H_2_O_2_ to mediate RDR1 induction, playing a critical role in restricting virus systemic infection and accumulation (Liao et al., 2013). In the present study, SA pretreatment induced *SlRDR1a* expression rapidly, and this downstream response enhanced the defence against PSTVd infection in tomato plants (Figure 6). In contrast, SA did not increase *SlRDR1a* transcript abundance in *SlRDR1a*‐silenced plants and, interestingly, the resistance of *SlRDR1a*‐silenced tomato to PSTVd was partially restored by exogenous SA application (Figure 6). This suggests that SA might regulate multiple pathogen defence pathways in tomato, such as other silencing‐related genes like *DCL1*, *DCL2*, *RDR1*, and *RDR2* in tomato that are induced after SA treatment (Campos et al., 2014) or genes related to an AOX‐associated defence pathway (Liao et al., 2014). Thus, further experiments to determine the other factors involved in SA‐associated induction will reveal the mechanisms of plant antiviroid defence reactions.

In summary, we provided evidence of crosstalk between RDR1 and SA in response to viroid infection, and preliminarily revealed the molecular mechanisms of RDR1 function during viroid infection, which increases our understanding of the plant mechanisms involved in defence responses against viroid attack and provides theoretical support for the development of technology to control viroid diseases.

## 4 EXPERIMENTAL PROCEDURES

### 4.1 Plant materials and growth conditions

*N*. *benthamiana* seedlings transformed with pCAM‐1300 empty vector (EC), *N*. *benthamiana* transformed with *NtRDR1* (GenBank accession no. AJ011576), *N*. *benthamiana* transformed with *CsRDR1c1* (GenBank accession no. KT316426) from cucumber, and tomato plants cv. Rutgers were grown in an insect‐free greenhouse with supplementary lighting. The temperature was maintained at 28 ℃ (day) and 25 ℃ (night) with a 16/8 hr (light/dark) photoperiod and 80% relative humidity. Seedlings at the four‐ to six‐leaf stage were used for viroid inoculation. *N*. *benthamiana* (NtRDR1) transgenic seedlings were kindly provided by Professor Huishan Guo at the Institute of Microbiology, Chinese Academy of Sciences (Ying et al., 2010). *N*. *benthamiana* (EC) and *N*. *benthamiana* (*CsRDR1c1*) transgenic experiments were performed by Wuhan Boyuan Biological Co., Ltd.

### 4.2 Plasmid construction and inoculation

PSTVd‐s (GenBank accession no. MK303581) was used in our experiments. pGEM‐PSTVd plasmid contained head‐to‐tail tandem PSTVd cDNA repeats. The linearized fragment of pGEM‐PSTVd was digested using *S* *pe*I (Takara), and then the PSTVd dimer RNA plus‐strand was transcribed using T7 RNA polymerase (Promega). The transcribed PSTVd dimer RNA was then digested using RNase‐free DNase I (Takara) to remove the DNA template. Finally, the RNA concentration was adjusted to 100 ng/µl in 100 mM sodium phosphate buffer (pH 7.5). Seedlings were inoculated with either PSTVd or buffer only. The third and fourth true leaves of each seedling were dusted with carborundum and rubbed evenly with 20 µl of viroid inoculum or sodium phosphate buffer; eight seedlings were used in each treatment. Inoculated plants were rinsed with distilled water, placed in the dark overnight, and then kept in the greenhouse under the same environmental conditions for 2–4 weeks (Xia et al., 2017). The full‐length PSTVd‐s dimer fragment was cloned into vector pCAM2300 between the *E* *co*RI/*B* *am*HI sites to generate the pCAM2300‐PSTVd infectious clone, which was transformed into *A*. *tumefaciens* GV3101 and used to infiltrate plant seedlings. The full length of *CsRDR1c1* was PCR amplified from a cDNA from a total RNA sample isolated from cucumber (cv. Suyo) leaf tissues. The 3′ terminus of *CsRDR1c1* was fused with the *GFP* gene through an overlap PCR to generate *CsRDR1c1‐GFP*. The full‐length *CsRDR1c1‐GFP* was then inserted into pCAM‐1300 vector between the *E* *co*RI and *B* *am*HI sites to generate pCAM1300‐CsRDR1c1‐GFP, which was transformed into *A*. *tumefaciens* GV3101 and used for genetic transformation. The primers used are shown in Table S1.

The VIGS constructs were generated following the method of Liao et al. (2014). A fragment of the coding region of *SlRDR1a* (approximately 400 nt) was cloned into vector pTRV2 between the *E* *co*RI and *B* *am*HI sites to generate pTRV2:SlRDR1a. *SlRDR1a* was amplified based on the sequence of the tomato gene (*SlRDR1a*; Solyc05g007510.2.1) in the International Tomato Annotation Group (ITAG) database. pTRV1 (TRV‐RNA1) and pTRV2:SlRDR1a were transformed into *A*. *tumefaciens* and used to infiltrate tomato seedlings. A fragment of *Sl* *PDS* (412 nt) was also inserted into pTRV2 (pTRV2:SlPDS), serving as a positive control; the negative control for the agroinfiltration of tomato seedlings was the pTRV2 empty vector (pTRV2:EV). VIGS analysis was performed by coinfiltrating a mixture of pTRV1‐ and pTRV2‐carrying *A*. *tumefaciens* suspensions into the bottom leaves of 12‐day‐old tomatoes according to Liu et al. (2002). After viral infection, the plants were maintained in a greenhouse (26 ℃) before use. PCR fragments were amplified using the primers shown in Table S1. All constructs were confirmed by sequencing and then transformed into *A*. *tumefaciens*. For exogenous SA treatment, tomato plants were sprayed to run‐off with 2 mM SA (Johnson Matthey Catalog Company, Inc.) in buffer (sterile water with 0.05% Tween 20) or buffer control (Zhang et al., 2016).

### 4.3 RT‐PCR and RT‐qPCR

Total RNAs were extracted from *N*. *benthamiana* and tomato plant systemic leaves (14, 21, and 28 dpi) using the cetyltrimethylammonium bromide (CTAB) method (Zhang et al., 2012). The RNA quantity and concentration were assessed using agarose gel electrophoresis and a NanoDrop 2000 spectrophotometer (NanoDrop Technologies). Reverse transcription was performed using M‐MLV reverse transcriptase (Promega) with random primers (Sangon Biotech) at 37 ℃ for 1 hr then 72 ℃ for 10 min. The genomes of PSTVd were amplified using KOD FX Neo high‐fidelity DNA polymerase (TOYOBO) using the specific primers PSTVd‐F/PSTVd‐R. To analysis the 72‐nt insert of *RDR1* mRNA in *N*. *benthamiana*, total cDNA from *N*. *benthamiana* and *N*. *benthamiana‐*NtRDR1 was amplified using primers NbRdR1m/NtRDR1‐F (1315–1335) and NbRdR1m/NtRdR1‐R (1851–1870) according to the sequence alignment of a portion of *N*. *tabacum* *NtRDR1* and *N*. *benthamiana* *NbRdR1m* (GenBank accession no. AY574374) (Yang et al., 2004). The PCR products were cloned into vector pTOPO‐blunt (Aidlab) according to the manufacturer's instructions, followed by transformation into *Escherichia coli* DH5α cells (TransGen Biotech), and the positive clones were selected and sequenced (Sangon Biotech). For RT‐qPCR analysis, total RNAs from systemic leaves of inoculated plants were extracted using an RNAprep pure polysaccharide polyphenols total RNA extraction kit (Tiangen Biotech) according to the manufacturer's protocol. PSTVd genomic RNA, *SlRDR1a* mRNA, and the TRV capsid protein coding gene (*cp*) levels in plant samples were analysed by RT‐qPCR. Total RNA (1 µg) from each sample was used for cDNA synthesis by M‐MLV reverse transcriptase with random hexamer primers. Reactions without template were included as controls. The PCR was performed using a MyGo Pro Real Time PCR System (IT‐IS Life Science Ltd) using GoTaq qPCR Master Mix containing SYBR R Green I (Promega) as instructed, in a PTC‐200 thermal cycler (MJ Research/Bio‐Rad). The *S. lycopersicum* actin gene (*SlActin*) and *N*. *benthamiana* phosphatase 2A gene (*NbPP2A*) were used as internal reference genes for normalization of expression levels. The relative level of gene expression and virus accumulation was calculated using the ΔΔ*C*_t_ method. The efficiencies of the primers used for RT‐qPCR were calculated and had the appropriate values (Table S2).

### 4.4 Northern blotting and siRNA‐blot hybridization

Total sRNAs were extracted using an miRcute miRNA Isolation Kit (Tiangen). Northern blotting was performed as previously described (Zhang et al., 2014). In brief, DIG‐labelled cRNA probes for PSTVd RNA were obtained by in vitro transcription using a DIG RNA labelling kit (Roche Applied Science) according to the manufacturer's instructions. Total RNA of each sample was fractionated using 1.5% agarose gel electrophoresis (for genomic RNA) or 17% denaturing polyacrylamide gel (for sRNAs). The fractionated RNAs in the gel were transferred to positively charged Hybond‐N+ nylon membranes (Amersham) and immobilized by UV cross‐linking (1,200 × 100 J/cm^2^). Prehybridization was performed at 68 ℃ for 1 hr with PerfectHyb Plus hybridization buffer (Sigma), hybridization was performed overnight at 68 ℃ (genomic RNA) or 50 ℃ (sRNAs) with a DIG‐labelled cRNA probe. Hybridization was visualized by incubating the membrane with an alkaline phosphatase‐labelled anti‐DIG antibody and the disodium 3‐(4‐methoxyspiro[1,2‐dioxetane‐3,2′‐(5′‐chloro)tricyclo[3.3.1.1(3,7)]decan]‐4‐yl) phenyl phosphate (CSPD) chemiluminescence substrate (Roche Applied Science). Equal loading was confirmed by 5S rRNA fluorescence after ethidium bromide staining and UV irradiation (Tanon 2500).

### 4.5 Protein extraction and western blotting hybridization

Protein extraction and western blot hybridization were performed according to Li et al. (2018). After transferring protein to nitrocellulose membranes, the membranes were probed with a mouse anti‐GFP or anti‐myc antibody and then probed with a horseradish peroxidase‐labelled goat anti‐mouse antibody. Detection signal was visualized using the EasySee Western Blot Kit (TransGen Biotech) according to the manufacturer's protocol.

### 4.6 Deep sequencing and sequence analysis of viroid derived‐sRNAs

Uninoculated systemic leaves collected at 21 days after PSTVd inoculation were collected for sRNA sequencing. sRNA samples were prepared use TruSeq Small RNA Sample Prep Kits (Illumina) and sequenced using an Illumina Hiseq 2000/2500 instrument; the sequencing read length was 1 × 50 bp. ACGT101‐miR (LC Sciences) was used to remove adapter dimers, junk, low complexity, common RNA families (rRNA, tRNA, snRNA, snoRNA), and repeats from the raw reads. The project was carried out by Lc‐Bio Technologies (Hangzhou) Co., Ltd. The obtained sRNA sequences (21–24 nt) were mapped to the PSTVd genome, and the circularity of the viroid genome was taken into consideration. For further analysis, the 21–24 nt sequences were pooled, and each set of sequences was analysed by BLAST searching against the nucleotide sequence of the PSTVd‐s strain. No mismatch was allowed. Data were analysed and visualized for specific distribution patterns and phasing (WPS Office Excel 2019).

### 4.7 Data analysis

All data were analysed using Student's *t* test (*n* = 6), with three independent replicates in each experiment. In the figures, ** indicates statistically significant differences compared with the control at *p* < 0.01 and different letters indicate significant differences between treatments (*p* < 0.05).

## CONFLICT OF INTEREST

There is no conflict of interest to declare.

## Supporting information

**FIGURE S1** RT‐PCR analysis of *RDR1* mRNA sequences for the 72‐nt insert. DNA maker shows size‐marker fragments representing 250, 500, and 750 bp (bottom to top). The EC samples contained a single amplicon (lines 1–6), whereas the *NtRDR1* samples contained two amplicons that differed by approximately 72 nt (lines 7–12)

**FIGURE S2** Accumulation analysis of PSTVd in CsRDR1c1 and EC transgenic lines. Northern blot detection with digoxigenin (DIG)‐labelled PSTVd complementary RNA (cRNA) probe in CsRDR1c1 and EC transgenic lines at 14 and 28 days postinoculation (dpi). As a loading control, ribosomal RNAs (rRNAs) were stained with ethidium bromide. The two bottom panels correspond to the western blot anti‐GFP and the protein loading control in CsRDR1c1 and EC transgenic lines at 28 dpi, respectively

**FIGURE S3** Sequence alignment analysis of the *Nicotiana tabacum* (tobacco) NtRDR1, *Cucumis sativus* (cucumber) CsRDR1c1, and *Solanum* *lycopersicum* (tomato) SlRDR1a proteins. GenBank accession numbers are as follows: NtRDR1: AJ011576; SlRDR1a: NM_001247390.1 (Solyc05g007510.2.1); CsRDR1c1: KT316426. The amino acid positions of each conserved structure of RdRP are as follows: NtRDR1‐RdRP: 362–937 amino acids; CsRDR1c1‐RdRP: 73–935 amino acids; SlRDR1a‐RdRP: 362–935 amino acids. (a) and (b) Sequence similarity alignment use conserved structure of RdRP indicated that NtRDR1‐RdRP shares 88.5% and 70.3% identical amino acid sequence with SlRDR1‐RdRP and CsRDR1c1‐RdRP, respectively. Sequence alignment were performed with the use of the DNAMAN program

**FIGURE S4** Semiquantitative RT‐PCR analysis showing the effect of VIGS on SlRDR1a transcription. Ethidium bromide‐stained agarose gels showing RT‐PCR products. The first‐strand cDNA was generated from total RNA isolated from silenced and nonsilenced plants using an oligo(dT) primer. cDNA samples were then used for PCR amplification using *SlRDR1a‐* and *SlActin*‐specific primers. (a) PCR products for *SlRDR1a* (left) and *SlActin* (right) derived from TRV alone infected tomato plants. (b) PCR products for *SlRDR1a* (left) and *SlActin* (right) derived from *SlRDR1a*‐silenced tomato plants. Lanes 1–7 correspond to products from PCR cycle number 15, 18, 21, 24, 27, 30, and 33. Lane 8 represents the control, in which the RT reaction mix without reverse transcriptase was used as a template in the reaction. M represents DNA marker

**FIGURE S5** PSTVd induced the expression of *SlRDR1a* in tomato plants. RNA samples were collected from the upper uninoculated leaves of tomato plants 4 days after agroinfiltration with pCAM2300‐PSTVd in the lower leaves and subjected to a quantitative reverse transcription PCR assay to evaluate the *SlRDR1a* mRNA in tomato plants. The results are expressed as the mean ± *SD* based on three biological replicates, *n* = 6. **p < .01 compared with the control using Student’s *t* test

**TABLE S1** Primers used in this study

**TABLE S2** Overall features of potato spindle tuber viroid (PSTVd)‐small RNAs (sRNAs) isolated from *Nicotiana benthamiana* plants infected with PSTVd at 21 days postinoculation

## Data Availability

The data that support the findings of this study are available from the corresponding author upon reasonable request.
