# Supplementary material for: RNA‐dependent RNA polymerase 1 delays the accumulation of viroids in infected plants
Source: Mol Plant Pathol. 2021 Jul 23;22(10):1195–208. doi: 10.1111/mpp.13104 (PMC8435232; doi:10.1111/mpp.13104)
Supplement: Supplementary file 1 — FIGURE S1 RT‐PCR analysis of RDR1 mRNA sequences for the 72‐nt insert. DNA maker shows size‐marker fragments representing 250, 500, and 750 bp (bottom to top). The EC samples contained a single amplicon (lines 1–6), whereas the NtRDR1 samples contained two amplicons that differed by approximately 72 nt (lines 7–12) [file MPP-22-1195-s007.docx]

**FIGURE S1 RT-PCR analysis of RDR1 mRNA sequences for the 72-nt insert.**

DNA maker shows size-marker fragments representing 250, 500, and 750 bp (bottom to top). The EC samples contained a single amplicon (line 1-6) whereas the NtRDR1 samples contained two amplicons that differed by approximately 72 n (line 7-12).


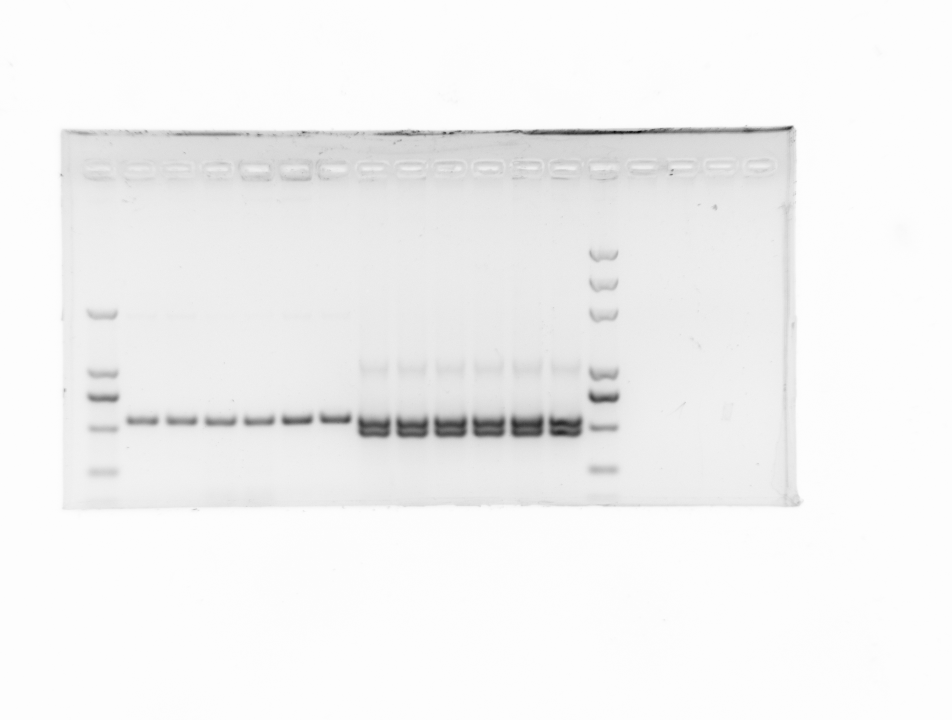


**M 1 2 3 4 5 6 7 8 9 10 11 12**
