# Supplementary material for: RNA‐dependent RNA polymerase 1 delays the accumulation of viroids in infected plants
Source: Mol Plant Pathol. 2021 Jul 23;22(10):1195–208. doi: 10.1111/mpp.13104 (PMC8435232; doi:10.1111/mpp.13104)
Supplement: Supplementary file 2 — FIGURE S2 Accumulation analysis of PSTVd in CsRDR1c1 and EC transgenic lines. Northern blot detection with digoxigenin (DIG)‐labelled PSTVd complementary RNA (cRNA) probe in CsRDR1c1 and EC transgenic lines at 14 and 28 days postinoculation (dpi). As a loading control, ribosomal RNAs (rRNAs) were stained with ethidium bromide. The two bottom panels correspond to the western blot anti‐GFP and the protein loading control in CsRDR1c1 and EC transgenic lines at 28 dpi, respectively [file MPP-22-1195-s002.docx]

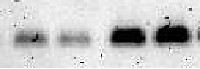

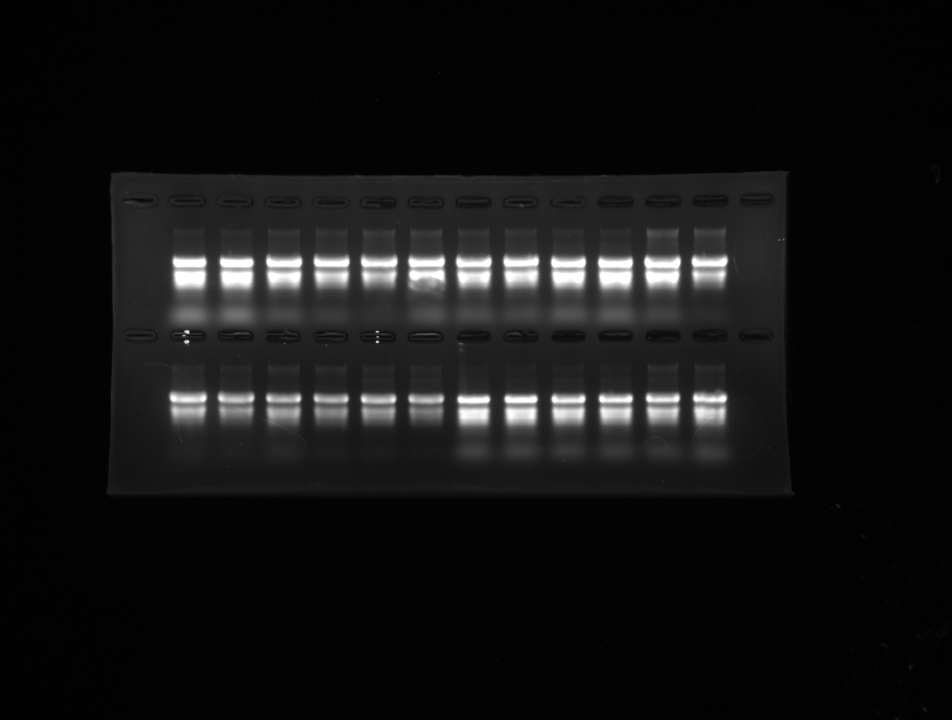

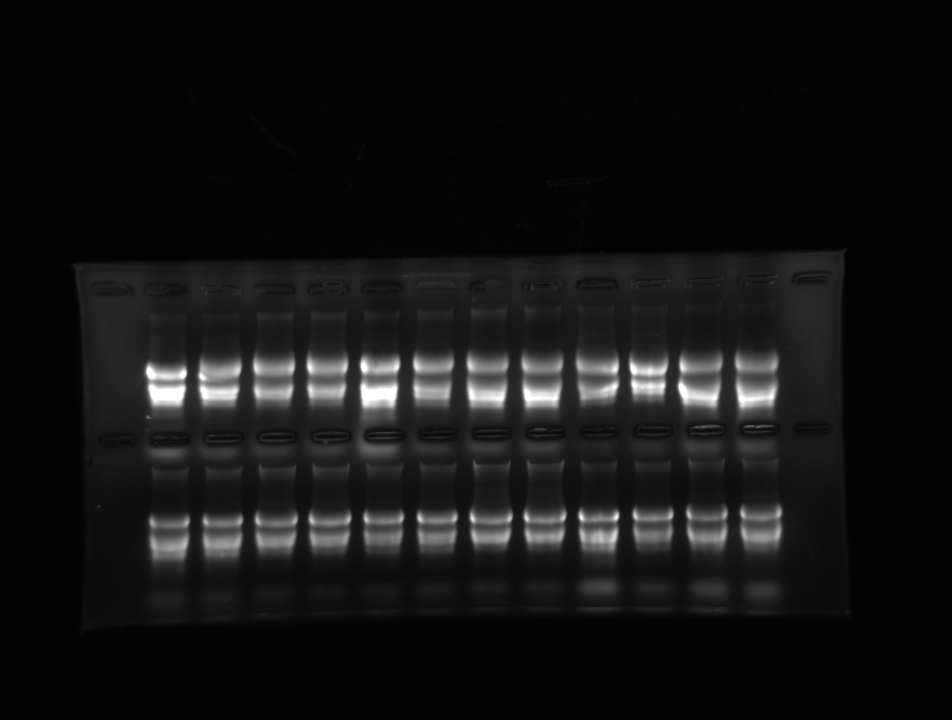


**EC**

**CsRDR1c1**

**PSTVd**

**rRNA**

**14 dpi**

**28 dpi**

**Anti-GFP**

**Loading**

**PSTVd**

**rRNA**


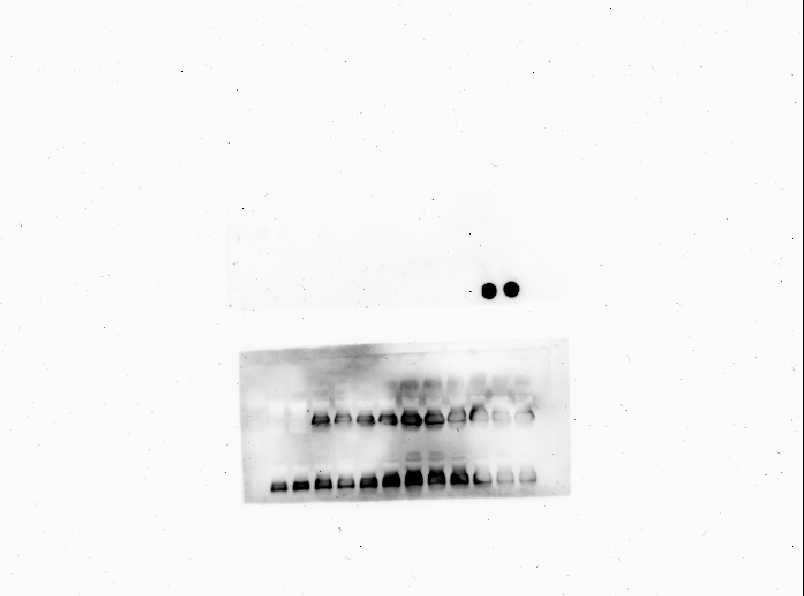

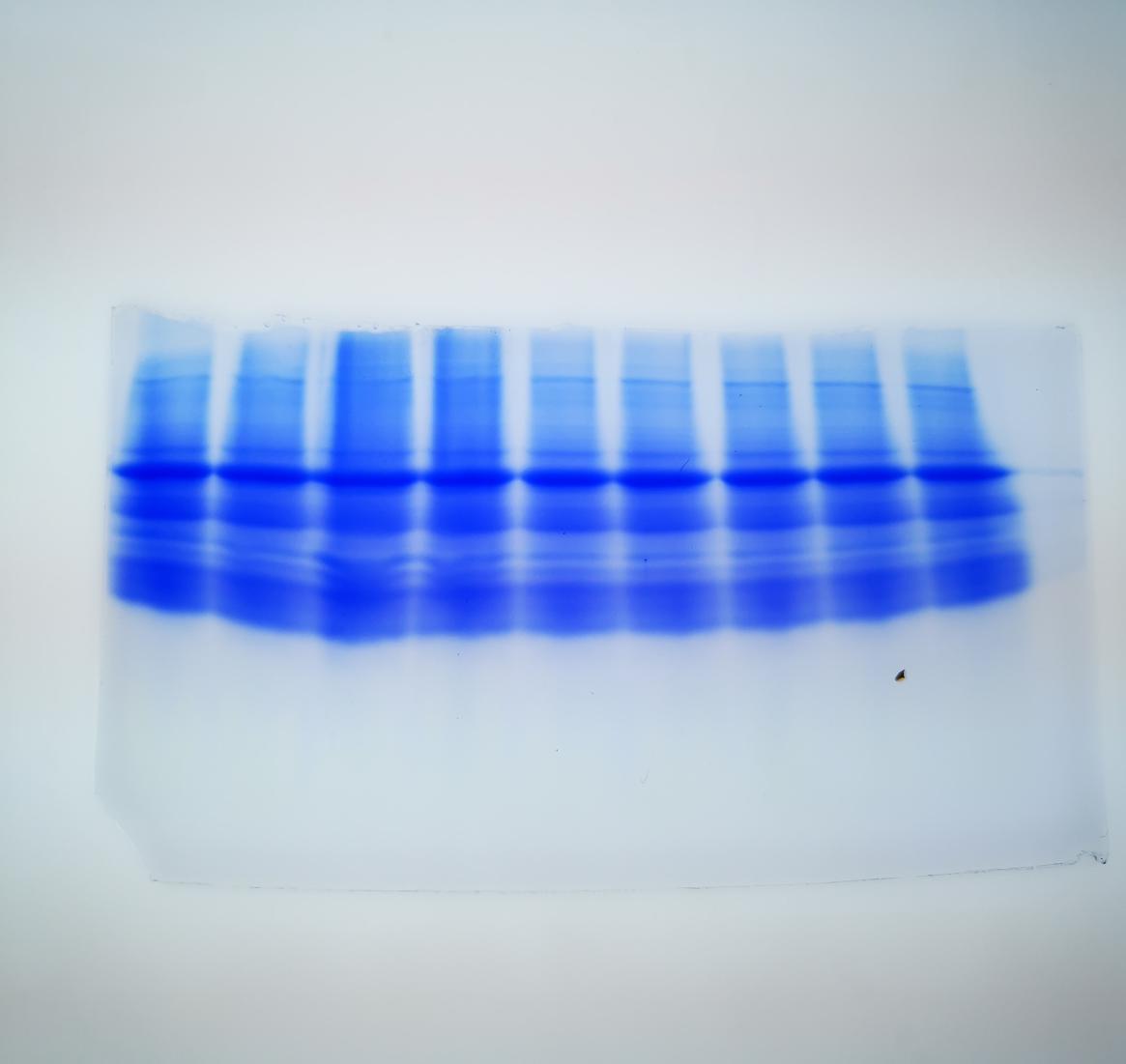


**28 dpi**


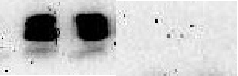


**FIGURE S2 Accumulation analysis of PSTVd in CsRDR1c1 and EC transgenic lines.**

Northern blot detection with digoxigenin (DIG)-labeled PSTVd complementary RNA (cRNA) probe in CsRDR1c1 and EC transgenic lines at 14 and 28 dpi. As a loading control, ribosomal RNAs (rRNAs) were stained with ethidium bromide. The two bottom panels correspond to the Western blot anti-GFP and the protein loading control in CsRDR1c1 and EC transgenic lines at 28 dpi, respectively.
