# Supplementary material for: RNA‐dependent RNA polymerase 1 delays the accumulation of viroids in infected plants
Source: Mol Plant Pathol. 2021 Jul 23;22(10):1195–208. doi: 10.1111/mpp.13104 (PMC8435232; doi:10.1111/mpp.13104)
Supplement: Supplementary file 3 — FIGURE S3 Sequence alignment analysis of the Nicotiana tabacum (tobacco) NtRDR1, Cucumis sativus (cucumber) CsRDR1c1, and Solanum lycopersicum (tomato) SlRDR1a proteins. GenBank accession numbers are as follows: NtRDR1: AJ011576; SlRDR1a: NM_001247390.1 (Solyc05g007510.2.1); CsRDR1c1: KT316426. The amino acid positions of each conserved structure of RdRP are as follows: NtRDR1‐RdRP: 362–937 amino acids; CsRDR1c1‐RdRP: 73–935 amino acids; SlRDR1a‐RdRP: 362–935 amino acids. (a) and (b) Sequence similarity alignment use conserved structure of RdRP indicated that NtRDR1‐RdRP shares 88.5% and 70.3% identical amino acid sequence with SlRDR1‐RdRP and CsRDR1c1‐RdRP, respectively. Sequence alignment were performed with the use of the DNAMAN program [file MPP-22-1195-s004.docx]

**a**


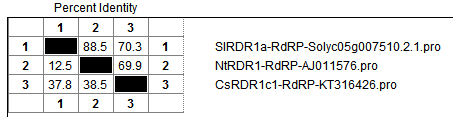


**b**

**
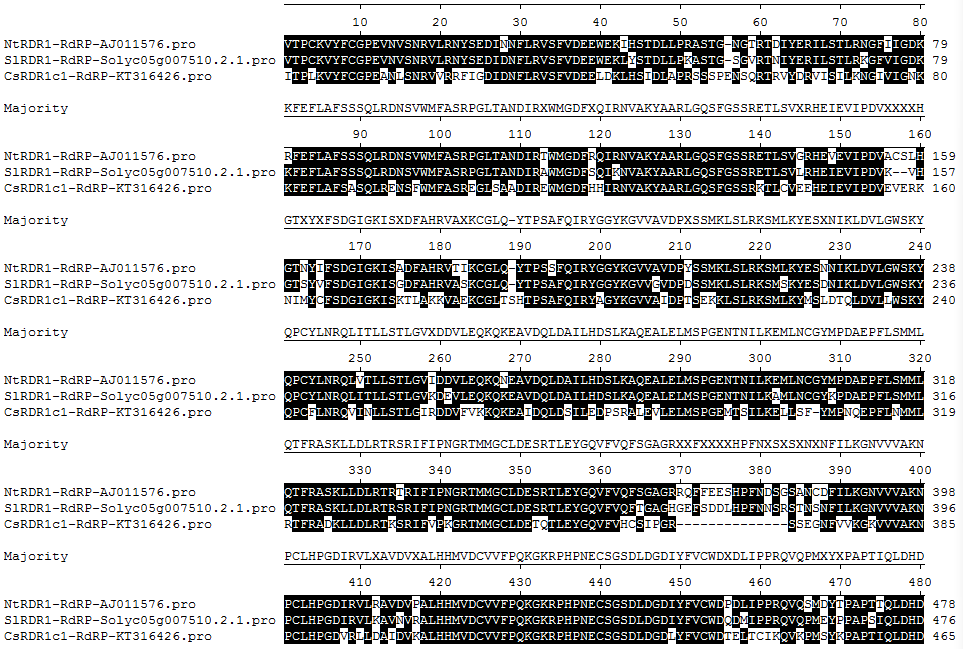
**

**FIGURE S3** Sequence alignment analysis of the *Nicotiana tabacum* (tobacco) NtRDR1, *Cucumis sativus* (cucumber) CsRDR1c1 and *Solanum lycopersicum* (tomato) SlRDR1a proteins. GenBank accession numbers are as follows: NtRDR1: AJ011576; SlRDR1a: NM_001247390.1 (Solyc05g007510.2.1); CsRDR1c1: KT316426. The amino acid positions of each conserved structure of RdRP are as follows: NtRDR1-RdRP: No. 362-937 aa; CsRDR1c1-RdRP: No. 373-935 aa; SlRDR1a-RdRP: No. 362-935 aa.

1. and (b) Sequence similarity alignment use conserved structure of RdRP indicated that NtRDR1-RdRP shares 88.5% and 70.3% identical amino acid sequence, with SlRDR1-RdRP and CsRDR1c1-RdRP, respectively. Sequence alignment were performed with the use of the DNAMAN program.
