# Supplementary material for: RNA‐dependent RNA polymerase 1 delays the accumulation of viroids in infected plants
Source: Mol Plant Pathol. 2021 Jul 23;22(10):1195–208. doi: 10.1111/mpp.13104 (PMC8435232; doi:10.1111/mpp.13104)
Supplement: Supplementary file 4 — FIGURE S4 Semiquantitative RT‐PCR analysis showing the effect of VIGS on SlRDR1a transcription. Ethidium bromide‐stained agarose gels showing RT‐PCR products. The first‐strand cDNA was generated from total RNA isolated from silenced and nonsilenced plants using an oligo(dT) primer. cDNA samples were then used for PCR amplification using SlRDR1a‐ and SlActin‐specific primers. (a) PCR products for SlRDR1a (left) and SlActin (right) derived from TRV alone infected tomato plants. (b) PCR products for SlRDR1a (left) and SlActin (right) derived from SlRDR1a‐silenced tomato plants. Lanes 1–7 correspond to products from PCR cycle number 15, 18, 21, 24, 27, 30, and 33. Lane 8 represents the control, in which the RT reaction mix without reverse transcriptase was used as a template in the reaction. M represents DNA marker [file MPP-22-1195-s003.docx]

**(a)**

**1 2 3 4 5 6 7 8 M 1 2 3 4 5 6 7 8**


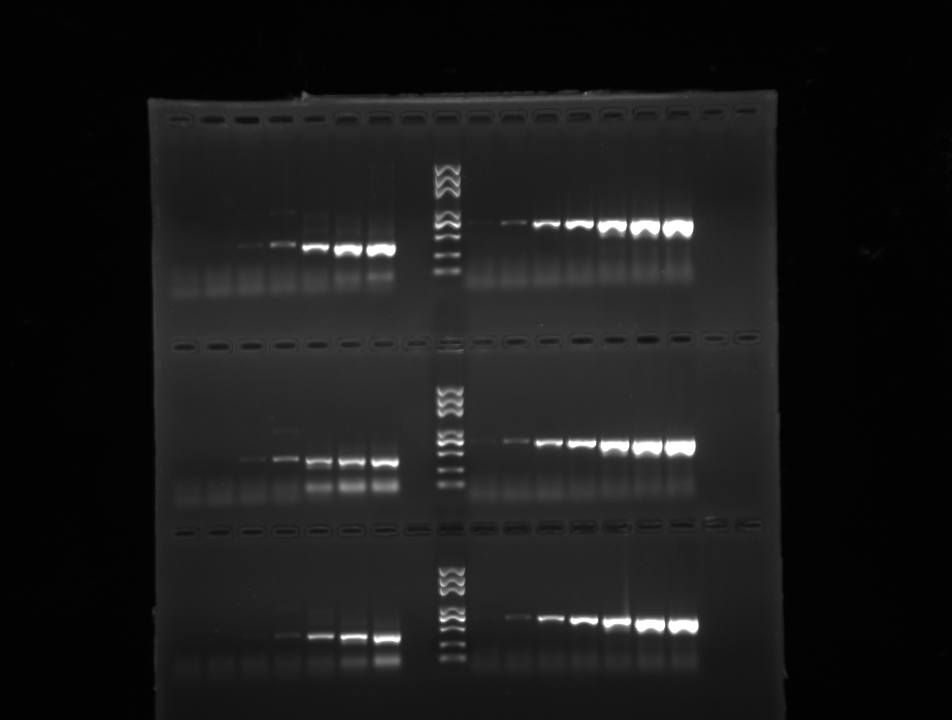

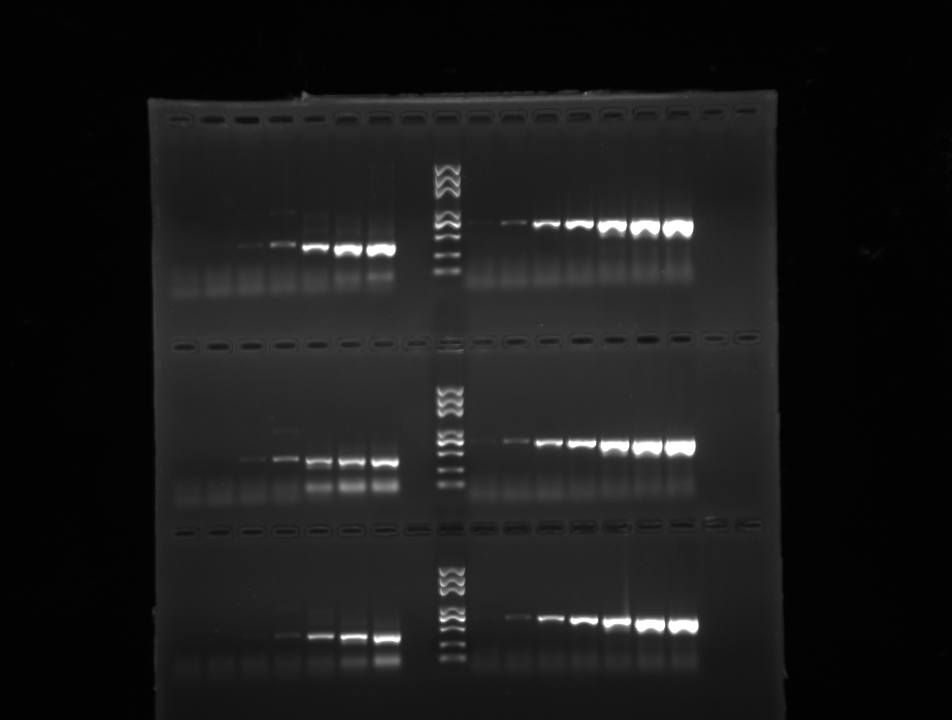


**1 2 3 4 5 6 7 8 M 1 2 3 4 5 6 7 8**

**(b)**

**FIGURE S4 Semi-quantitative RT-PCR analysis showing the effect of VIGS on SlRDR1a transcription.**

Ethidium bromide-stained agarose gels showing RT-PCR products. The first strand cDNA was generated from total RNA isolated from silenced and non-silenced plants using an oligo (dT) primer. Then used in a PCR reaction using SlRDR1a and SlActin specific primers. (a) PCR products for SlRDR1a (left) and SlActin (right) derived from TRV alone infected tomato plants. (b) PCR products for SlRDR1a (left) and SlActin (right) derived from SlRDR1a silenced tomato plants. Lanes 1-7 correspond to products from PCR cycle number 15, 18, 21, 24, 27, 30 and 33. Lane 8 represents the control, in which the RT reaction mix without reverse transcriptase was used as a template in the reaction. M represents DNA marker.
