# Supplementary material for: RNA‐dependent RNA polymerase 1 delays the accumulation of viroids in infected plants
Source: Mol Plant Pathol. 2021 Jul 23;22(10):1195–208. doi: 10.1111/mpp.13104 (PMC8435232; doi:10.1111/mpp.13104)
Supplement: Supplementary file 5 — FIGURE S5 PSTVd induced the expression of SlRDR1a in tomato plants. RNA samples were collected from the upper uninoculated leaves of tomato plants 4 days after agroinfiltration with pCAM2300‐PSTVd in the lower leaves and subjected to a quantitative reverse transcription PCR assay to evaluate the SlRDR1a mRNA in tomato plants. The results are expressed as the mean ± SD based on three biological replicates, n = 6. **p < .01 compared with the control using Student’s t test [file MPP-22-1195-s006.docx]

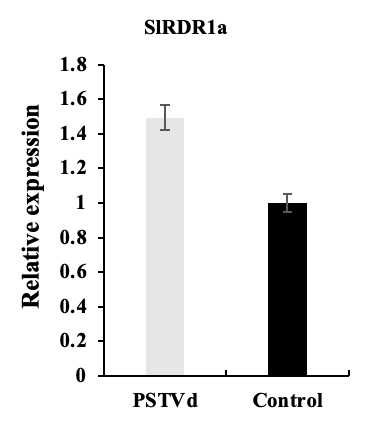


**

**FIGURE S5 PSTVd induced the expression of SlRDR1a in tomato plants.**

RNA samples were collected from the upper uninoculated leaves of tomato plants post 4 days agro-infiltrated with pCAM2300-PSTVd in the lower leaves, and subjected to an RT-qPCR assay to evaluate the SlRDR1a mRNA in tomato plants. The results are expressed as the mean ± SD based on three biological replicates, n = 6. **p < 0.01 compared with the control using Student’s t-test.
