# Supplementary material for: RNA‐dependent RNA polymerase 1 delays the accumulation of viroids in infected plants
Source: Mol Plant Pathol. 2021 Jul 23;22(10):1195–208. doi: 10.1111/mpp.13104 (PMC8435232; doi:10.1111/mpp.13104)
Supplement: Supplementary file 6 — TABLE S1 Primers used in this study [file MPP-22-1195-s005.docx]

| **Table S1 Primers used in this study** | | | | |
| --- | --- | --- | --- | --- |
| **Gene** | **Encoding protein** | **Accession No** | **Primer pairs** | **Note** |
| *Slactin* | *Solanum lycopersicum* Actin | U60481 | F: 5’-TGGTCGGAATGGGA  CAGAAG-3’  R: 5’-CTCAGTCAGGAGAA  CAGGGT-3’ | qPCR analyse  internal marker gene |
| *SlRDR1a* | *Solanum lycopersicum*  RNA-dependent RNA Polymerase1 | NM_001247390 | F: 5’-TGCATCCTGGTGAT  ATTCGT-3’  R: 5’-TCCAAATCACTCCC  AGAACA-3’ | qPCR analyse |
|  |  |  | F: 5’-GAGCCCTTTCTTTCA  ATGATG-3’  R: 5’-TCCAAATCACTCCC  AGAACA-3’ | VIGS tests  (2201-2601 bp) |
| *SlPDS* | *S. lycopersicum*  phytoene desaturase (*pds*) | M88683 | F: 5’-AGGCACTCAACTTTA  TAAACC -3’  R: 5’-GTTCTTCAGTTTTCT  GTCAAACC-3’ | VIGS tests  (1174-1586 bp) |
| *CsRDR1c1* | *Cucumis sativus*  RNA-dependent RNA Polymerase1 c1 | KT316426 | F: 5’-ATGGGAAAGACGAT  CGAA-3’  R: 5’-CCCCATCAAAATTG  TGTG-3’ | Over-expression |
| NbRDR1/NtRDR1 | Tobacco RNA-dependent RNA Polymerase1 | AY574374/  AJ011576 | F: 5’-CAAGAGCTAGTACT  GGAAATG-3’(1315-1335)  R: 5’-GAATATGGATCAAC  AGCCAC-3’ (1851-1870) | 72-nt insert of RDR1 mRNA in *N. benthamiana* |
| PSTVd-s strain | - | MK303581 | F: 5’-CCCTTCCTTTCTTCG  GGTGT-3’  R: 5’-TTGTTTCCACCGGG TAGTAGC-3’ | qPCR analyse |
| *NbPP2A* | *Nicotiana benthamiana*  Protein phosphatase 2A | X97913 | F: 5’-TGGCTCTTGACTAC  GAGCAGGAGCTT-3’  R: 5’-ACCACTGAGCACAA  TGTTACCGTAGAGGT-3’ | qPCR analyse  internal marker gene |
| HSVd-g54 strain | *-* | AB219944  (HSVd-g) | F: 5’-TCTTTGCTTGCCTGA  TGC-3’  R: 5’-AAACAAGGCAGGA  AGGTACT-3’ | A single A/G substitution at position 54 with respect to HSVd-g |
|  |  |  | F: 5’-GGAAGGTACTTACC  TGAGAAAGG-3’  R: 5’-CCAGGAGAAGGTAA  A GAAGAAGG-3’ | qPCR analyse |
| TRV-*cp* | *Tobacco Rattle Virus* capsid protein | - | F: 5’-TCATTTGACAAGTC  GGGC-3’  R: 5’-TGTGTTTGGATTCGC  AGG-3’ | qPCR analyse |
| *CsRDR1c1-GFP* | *Cucumis sativus*  RNA-dependent RNA Polymerase c1 fused with green fluorescent protein | KT316426/AAB47998 | CsRDR1c1(*Bam*HI)-F:  5’-GGATCCATGGGAAAGACGATCGAAAT-3’  GFP-CsRDR1c1-R:  5’-TTCTTCTCCTTTACTCATTCACCCCATCAAAAT-3’  CsRDR1c1-GFP-F:  5’-ATTTTGATGGGGTGAATGAGTAAAGGAGAAGAA-3’  GFP (*Sal*I)-R: 5’-GTCGACTTTGTATAGTTCATCCATGCCA-3’ | Overlap PCR |
